# Synthesis and Evaluation of Gelatin–Chitosan Biofilms Incorporating Zinc Oxide Nanoparticles and 5-Fluorouracil for Cancer Treatment

**DOI:** 10.3390/ma17133186

**Published:** 2024-06-29

**Authors:** Viswanathan Kaliyaperumal, Srilekha Rajasekaran, Rajkumar Kanniah, Dhinakaraj Gopal, Ganeshraja Ayyakannu Sundaram, Alagarsamy Santhana Krishna Kumar

**Affiliations:** 1 Department of Chemistry, Saveetha School of Engineering, Saveetha Institute of Medical and Technical Sciences (SIMATS), Chennai 602105, India; srilekha200073@gmail.com (S.R.); nkrajkumar7@gmail.com (R.K.); 2 Translational Research Platform for Veterinary Biologicals, Centre for Animal Health Studies (CAHS), Tamil Nadu Veterinary and Animal Sciences University (TANUVAS), Chennai 600051, India; dirtrpvb@gmail.com; 3 Department of Biotechnology, Madras Veterinary College, Tamil Nadu Veterinary and Animal Sciences University (TANUVAS), Chennai 600051, India; 4 Department of Research Analytics, Saveetha Dental College and Hospitals, Saveetha Institute of Medical and Technical Sciences, Poonamallee High Road, Chennai 600077, India; asgchem84@gmail.com; 5 Department of Chemistry, National Sun Yat-sen University, No. 70, Lien-Hai Road, Gushan District, Kaohsiung 80424, Taiwan; 6 Faculty of Geology, Geophysics and Environmental Protection, AGH University of Krakow, Al. Mickiewicza 30, 30-059 Krakow, Poland

**Keywords:** gelatin film, chitosan film, ZnO nanoparticles, 5-fluorouracil, biofilm

## Abstract

In this study, a novel multifunctional biofilm was fabricated using a straightforward casting process. The biofilm comprised gelatin, chitosan, 5-fluorouracil (5-FU)-conjugated zinc oxide nanoparticles, and polyvinyl alcohol plasticized with glycerol. The 5-FU-conjugated nanoparticles were synthesized via a single-step co-precipitation process, offering a unique approach. Characterization confirmed successful drug conjugation, revealing bar-shaped nanoparticles with sizes ranging from 90 to 100 nm. Drug release kinetics followed the Korsmeyer–Peppas model, indicating controlled release behavior. Maximum swelling ratio studies of the gelatin–chitosan film showed pH-dependent characteristics, highlighting its versatility. Comprehensive analysis using SEM, FT-IR, Raman, and EDX spectra confirmed the presence of gelatin, chitosan, and 5-FU/ZnO nanoparticles within the biofilms. These biofilms exhibited non-cytotoxicity to human fibroblasts and significant anticancer activity against skin cancer cells, demonstrating their potential for biomedical applications. This versatility positions the 5-FU/ZnO-loaded sheets as promising candidates for localized topical patches in skin and oral cancer treatment, underscoring their practicality and adaptability for therapeutic applications.

## 1. Introduction

The creation of new biodegradable films is critical for tissue engineering, wound treatment, and tailored surface coatings. Researchers have recently shown increased interest in biofilm-based cell seeding and tissue formation studies. Natural biomaterials, such as collagen, fibrin, agarose, alginate, hyaluronan, and chitosan, as well as synthetic biomaterials like polyethylene glycol and poly(lactic-co-glycolic acid), can now be used to form biofilms in tissue engineering [1,2,3,4]. For biological applications, the biofilm must be biocompatible, and the cells must maintain a high level of vitality. Following implantation, the biofilm must elicit a negligible immune response to prevent initiating an inflammatory reaction that could delay healing or cause rejection by the body [5,6].

Several varieties of biofilms have recently been developed, and their structural designs are largely based on fabrication processes, biocompatibility, biodegradability, cell adhesion, proliferation, and cell differentiation [7,8,9,10]. Additionally, the generated films can act as delivery vehicles and regulate the activation of cellular components. Because of their porous nature, these films also serve as carriers for nutrients and metabolites. Researchers have created biofilms using a range of techniques, including rapid prototyping, solvent casting, freeze drying, melting, and phase separation [11,12].

5-fluorouracil (5-FU) is a well-known anticancer agent, also known as an anti-neoplastic drug. The fluorinated pyrimidine in 5-FU molecules inhibits thymidylate synthetase, hence inhibiting RNA and DNA synthesis. 5-FU is mostly used to treat gastrointestinal and breast cancers. 5-FU therapy can cross the blood–brain barrier and is highly effective against brain cancer. Previously, topical creams containing 5 -FU were shown to treat actinic keratosis, basal cell carcinoma, and nail psoriasis [13,14,15]. The film-based formulations enabled simple and site-specific drug administration in topical applications. The drug delivery technology based on biodegradable skin films has a lengthy retention duration before re-releasing the active component, lowering diffusion barriers and enhancing medicine availability [16].

In this study, we constructed a biodegradable film incorporating nanomedicine. To create the biofilm, chitosan and gelatin were combined with polyvinyl alcohol and glycerol and then integrated with 5-fluorouracil (5-FU)-conjugated zinc oxide nanoparticles (ZnO NPs). This biofilm protects the nano-drug from leakage, ensuring controlled drug release and enhanced entry into target cells, which is crucial in cancer treatment as it improves therapeutic effects while also reducing side effects. The absence of preservatives and solvents in the film composition prevents skin irritation. The film’s anticancer activity was tested against a skin cancer cell line, and its functional characteristics were evaluated.

The combination of ZnO NPs and 5-FU in gelatin–chitosan biofilms represents a novel approach to cancer treatment. ZnO nanoparticles possess intrinsic anticancer properties and enhance drug delivery efficiency, while 5-FU is a well-established chemotherapeutic agent. Integrating these components into a biofilm matrix can yield synergistic effects, potentially leading to improved treatment outcomes. This study presents the preparation and evaluation of gelatin–chitosan biofilms containing ZnO NPs and 5-FU for cancer treatment. A systematic methodology was employed to explore the synthesis process, characterization techniques, and assessment of biofilm features, including drug release kinetics and anticancer activity. The unique combination of gelatin–chitosan, ZnO NPs, and 5-FU demonstrates significant potential for targeted cancer therapy, creating a versatile platform for localized drug delivery and tumor suppression.

## 2. Materials and Methods

### 2.1. Preparation of 5-Fluorouracil–β-Cyclodextrin/Zinc Oxide Nanoparticles 

For the preparation of 5-FU/ZnO NPs, a previously published procedure was modified to a single co-precipitation method [17]. In brief, 2.43 g of beta-cyclodextrin was mixed with 50 mL of Milli-Q water, 3 g of 5-fluorouracil powder was added, and the solution was stirred for 2 h at room temperature, followed by 11.84 g of zinc nitrate, 80 mL of KOH, and stirring for 30 min. The pellet was cleaned with ethanol twice and dried at 70 °C. The 5-Fu–β-CD/ZnO and β-CD/ZnO particles’ morphology, chemical composition, crystalline structure, and thermal properties were examined by employing TEM (S-3400 N model, Hitachi, Japan), SEM-EDX (Quanta 200 FEG), FT-IR (Perkin Elmer Spectrum1 FT-IR instrument), Raman spectra (Bruker RFS 27: Stand-alone FT-Raman Spectrometer), and XRD (Bruker) at the Sophisticated Analytical Instrumentation Facility (SAIF), Indian Institute of Technology, Chennai, using established methods. 

### 2.2. The Phase Solubility Test Determines the β-CD Concentrations

For this study, 5 g of the 5-FU drug was mixed with 50 mL of a solution containing various concentrations of β-CD solutions (0, 3.125, 6.125, 12.50, 25,50, 100, and 200 mM), and then it was vigorously shaken at 25 °C for 48 h until equilibrium was attained. The samples were filtered through a 0.22 μm Millipore membrane filter and diluted to appropriate concentrations for determination. Three replicates were set for each treatment. The phase solubility graph was plotted using a concentration of 5-FU loaded on β-CD vs. the concentration of β-CD. The stability constant *Ks* was calculated based on the phase solubility diagrams following the below equation:

*Ks* = Slope/*S*0 (1 − Slope)

where slope represents the slope of the phase solubility diagram, and *S*0 is the 5-FU equilibrium concentration.

### 2.3. Optimizing Drug Loading Quantity

A different drug concentration of 5-FU was added to 50 mL of Milli-Q water containing 2.4 g of β-CD, followed by 11.84 g of zinc nitrate and 80 mL of KOH. The mixture was stirred at room temperature, while the supernatant was used for drug loading equilibrium testing. A spectrometer was used to evaluate the drug encapsulation efficiency of 5-FU at a maximum of 265 nm. The following formula was used to compute the encapsulation efficiency: Maximum Encapsulation Efficiency (EE) = [(Total amount of 5-FU − Amount of free 5-FU in the supernatant)]/Total amount of 5-FU × 100.

### 2.4. Drug Release Studies In Vitro

The membrane diffusion method from our previous paper [18] was utilized to study the release of 5-FU from 5-FU/ZnO nanoparticles. To maintain the sink state, the nanoparticles were placed in a dialysis membrane (MW cut-off: 3.5 KDa), which was then placed in a beaker containing 100 mL of PBS pH 7.4 and 10% *v*/*v* methanol. The receptor medium was held at a constant temperature of 37± 0.5 °C and stirred with a magnetic stirrer. Regularly, aliquots of 5 mL samples were removed, and the same volume of medium was provided after each withdrawal. The samples were evaluated using a UV spectrophotometer set to 265 nm. The tests were carried out on duplicate samples.

### 2.5. Chitosan Preparation

Chitosan was dissolved in 0.5 M acetic acid and diluted with double-distilled water to achieve a 5% (*w/v*) solution. Subsequently, the chitosan solution was mixed with glycerol for 30 min.

### 2.6. 5-FU/ZnO/Gelatin–Chitosan Film

To commence, gelatin was dissolved in distilled water at 70 °C for 20 min, achieving a concentration of 5% (*w/v*). PVA (5% *w/v*) was dissolved through magnetic stirring at 80 °C for 2 h. For the development of gelatin–chitosan films, a mixture comprising 100 mL of 5% gelatin, 60 mL of 5% PVA, 20 mL of 5% chitosan, 10 mL of glycerol, and 10 mL of 5-FU nanoparticles (1 g/mL) in milli-Q-water was prepared. This mixture was subjected to magnetic stirring and sonicated for 20 min in an ultrasonic cleaner bath. Subsequently, the film-forming solutions were poured into Petri dishes and left to dry at room temperature for 48 h. Upon completion of curing, the films were gently detached from the casting surface [18].

### 2.7. Cell Cytotoxicity Study

The A431 skin cancer cells were received from the National Centre for Cell Sciences (NCCS) in Pune and cultured in plastic tissue culture flasks in DMEM as a monolayer and suspension culture. The normal human fibroblast cells used in this investigation were obtained from Sigma and cultured in a DMEM mix containing calf serum and supplements (Gibco). The cell cytotoxicity studies were performed based on a previously published method [19,20]. To test cytotoxicity, 1 × 10^4^ cells/well were planted in 96-well plates using DMEM (Gibco, Thermofisher) medium and incubated in 5% CO_2_ at 37 °C for 24 h. After one hour, the medium was changed and incubated with plain chitosan, gelatin, chitosan–gelatin–chitosan–5-FU/ZnO nanoparticle-integrated films. The films were added to the wells and incubated for 24 h at 37 °C in a CO_2_ incubator. A total of 100 μL of MTT (Sigma) at a concentration of 5 mg/mL was applied to the wells containing varying quantities of cells and nanoparticles. It was incubated for 4 h at 37 °C. After removing the medium, 20 μL of DMSO (Sigma) was applied to the wells. After 15 min of incubation at 37 °C, the absorbance at 575 nm was measured.

## 3. Results

The current study developed a hybrid biofilm incorporating nanodrugs for cancer therapeutic applications. Actinic keratosis and superficial basal cell carcinoma are currently treated with 5-fluorouracil (5-FU) medications, as are various other dermatological conditions, such as squamous cell carcinoma in situ, warts, nail psoriasis, keratoacanthoma, and vitiligo. To address these dermatological issues, a slow-release, biodegradable nanomedicine-integrated film was developed. The combination of zinc oxide nanoparticles (ZnO NPs) and gelatin–chitosan not only protects the skin against sunburn but also modulates the entry of drug molecules into the infection site and surrounding tissue, reducing unwanted effects.

A simple precipitation process was used to generate the 5-FU-coated nanoparticles [21,22]. Beta-cyclodextrin (β-CD) was employed as a capping and stabilizing agent due to its ability to form complexes with drugs and zinc oxide molecules [21,22]. Its hydrophilic and lipophilic properties allow for multiple binding sites, enhancing the stability of the complex. The phase solubility test, measured at 264 nm, determined the equilibrium concentration of β-CD required to form an inclusion complex with 5-FU. The UV absorption value initially increased to 25 mM before plateauing at 50 mM, demonstrating that an inclusion complex could be generated using 50 mM β-CD with computed Ks values of 997 M^−1^.

**Figure 1:**
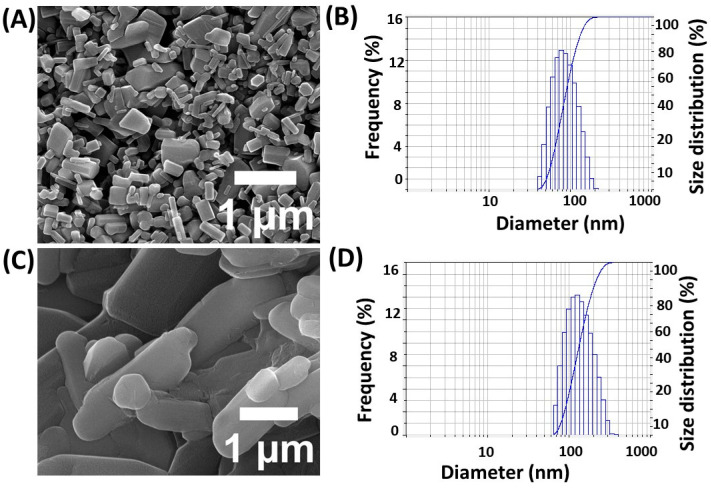
Physico-chemical characterization results of nanoparticles for SEM images of ZnO NPs (**A**), 5-FU/ZnO NPs (**C**), DLS spectra for (**B**,**D**), particle size analysis of ZnO NPs (**C**), and 5-FU/ZnO NPs (**D**).

The efficacy of drug encapsulation was initially investigated using five different 5-FU dosages [21,22]. This study found that 1 g of 5-FU is sufficient to achieve a maximum drug encapsulation efficiency rate of 84 ± 0.5%, while keeping β-CD and zinc nitrate concentrations constant. Additionally, the stability of the encapsulated drug was assessed over a period of time to ensure consistent release profiles. SEM and a dynamic light scattering (DLS) analyzer were used to determine the size and shape of the nanoparticles. The SEM images revealed that the generated particles were bar-shaped (Figure 1A), with average diameter values of 90–95 nm, as shown in Figure 1B. Dynamic light scattering confirmed particle sizes of approximately 90–100 nm, in agreement with the SEM findings. Furthermore, the zeta potential measurements indicated a stable colloidal suspension, enhancing the potential for drug delivery applications.

**Figure 2:**
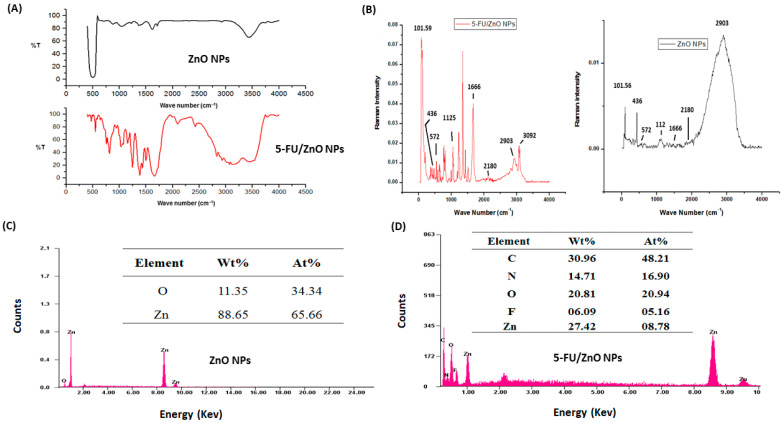
FT-IR, Raman, and EDX spectra of ZnO and 5-Fu/ZnO NPs. (**A**) FT-IR spectra analysis findings. (**B**) Raman spectra analysis results. (**C**) Analysis of EDX spectra-based ZnO nanoparticles and (**D**) 5-FU/ZnO nanoparticles.

Figure 2A shows the findings of determining surface functional groups using FT-IR spectroscopy. The FT-IR spectra of ZnO nanoparticles exhibited comparable results to previously published publications [22,23,24]. The FT-IR spectra revealed representative keys and evidence for the structural code of 5-FU-incorporated loaded nanoparticles. Significant bands were pointed out in the following region or range: 3480 cm^−1^ for hydroxyl (-OH) groups, 2925–2930 cm^−1^ for methyl groups (-CH), 1625–1655 cm^−1^ for amide stretching (−C=O), and 1275–1280 cm^−1^ for carbonyl (−CN) bonds. The O-H groups of β-CD stretching and deformation bands were detected at 3398 and 1154 cm^−1^, respectively. The peak at 2921 cm^−1^ confirmed the presence of a C-H group on the surface of the nanoparticles due to β-CD. At 654 cm^−1^, the Zn-O characteristic signal was found. Peaks at 1627, 1223, 1039, and 878 cm^−1^ represent HOH, C-O, C-O-C glucose units, and the C-O-C moiety of β-CD rings, respectively. The FT-IR spectra of 5-FU/ZnO nanoparticles revealed that the absorption bands at 1661.51, 1449.89, 3136.40, 1430.70, and 1246.87 cm^−1^ belong to the C=O, C=C, N-H, CF, and C-N stretching vibrations of 5-FU, respectively, while the peak at 1349.35 cm^−1^ corresponds to the pyrimidine compound vibration [23,24,25,26], confirming 5-FU. The peak of the Zn-O characteristic shifted from 654 to 642 cm^−1^. Figure 2B shows the results of a Raman spectroscopy study using ZnO and 5-FU/ZnO nanoparticles. The spectrum of ZnO nanoparticles revealed six modes: 436, 572, 1125, 1666, 2029, 2180, and 2903 cm^−1^. The Raman spectra rules [27] show that the produced nanoparticles are nonpolar and Raman-active, with the E_2_ and A_1_ (LO) modes recorded at 436 and 572 cm^−1^, respectively. The peak at 436 cm^−1^ appears narrower and sharper due to their high purity [27]. The 5-FU coating on ZnO nanoparticles resulted in a decrease in intensity at 436 cm^−1^ and 572 cm^−1^, as well as the development of new peaks.

The elemental composition and purity of ZnO and 5-FU/ZnO nanoparticles were evaluated using EDX spectra. Figure 2C, D demonstrate the results; the results supported the presence of a ZnO nanoparticle composed of Zn (88.65%) and O (11.35%). The EDX spectra of 5-FU/ZnO nanoparticles exhibited an extra C, N, and F signal caused by the medication.

**Figure 3:**
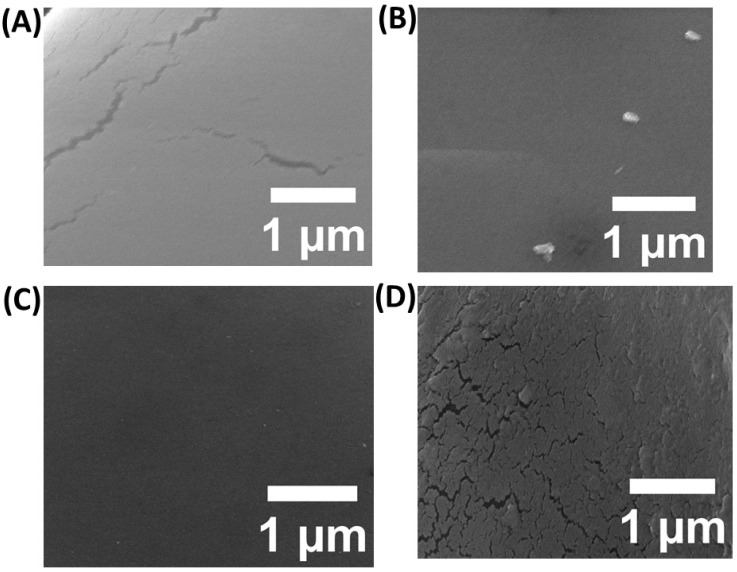
SEM confirmation of the (**A**) chitosan film, (**B**) gelatin film, (**C**) gelatin–chitosan hybrid, and (**D**) results from the gelatin–chitosan films containing 5-FU/ZnO.

The biodegradable film was made utilizing gelatin, chitosan, the film-casting ingredient PVA, and a plasticizer (glycerol). Initially, gelatin, chitosan, gelatine–chitosan, and gelatin–chitosan–5-Fu/ZnO NPs films were prepared and characterized individually using SEM, EDX, FT-IR, and Raman spectroscopy. The SEM images of the chitosan film showed a wave-like surface; gelatine showed a very smooth surface with air bubbles; gelatin–chitosan was smooth and dense; and after incorporating 5-FU/ZnO nanoparticles, the gelatine–chitosan film became slightly rougher and nanoparticle layers appeared, which could be attributed to the uniform dispersion of nanoparticles embedded in the gelatin and chitosan network structures. These photographs demonstrated good compatibility between the nanoparticles and the film formulation made with chitosan and gelatine, as shown in Figure 3.

**Figure 4:**
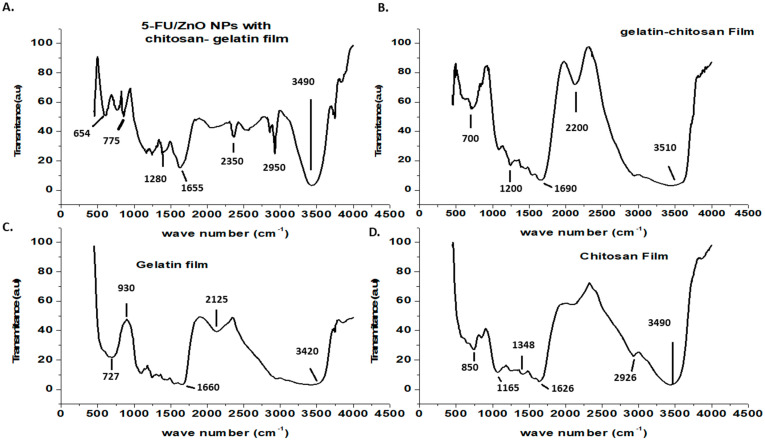
FT-IR-based confirmation of (**A**) results from gelatin–chitosan films containing 5-FU/ZnO. (**B**) Gelatin-chitosan hybrid gelatin film; (**C**) gelatin film; and (**D**) chitosan film findings.

The surface characterizations of the generated biofilms were investigated using FT-IR, and the results are shown in Figure 4. The FT-IR spectrum of chitosan shows a weak band at 2926 cm^−1^ due to C-H stretching, a band at 1626 cm^−1^ [24,25,26] due to secondary amide carbonyl C=O stretching, a bonding vibration of the N-H bond in non-acylated 2-aminoglucose primary amines, a band at 1384 cm^−1^ (CH_3_) symmetric deformation mode, and a N-H stretching of the amide band at 1165 cm^−1^ due to an oxygen bridge (C-O-C cyclic ether from the saccharide cycle) [24,25,26]. Chitosan saccharide structures have been identified in a minor peak at 850 cm^−1^ [24,26]. The gelatin film’s FT-IR spectra showed the characteristic amine functional group at 3420 cm^−1^ [24,26]. Other peaks in the gelatine film spectra were discovered at 2125 cm^−1^ (CAH stretching of alkenes) and 1662 cm^−1^ (amide-I, CO, and CN stretching) [24,25,26]. Other peaks found at 930 and 727 cm^−1^ were attributed to carboxylic acid CAO stretching and amine C=N stretching, respectively [24,25]. Peaks at 1690 and 1200 cm^−1^ were seen in the gelatin–chitosan hybrid film, resulting from peak shifts of gelatin and chitosan to lower wave numbers. This suggested that hydrogen bonds and hydrophobic interactions were the most important interactions between chitosan and gelatin [24,25]. The N-H bond in the non-acylated 2-aminoglucose primary amine group of chitosan at 1348 cm^−1^ disappeared in the hybrid film, indicating the formation of a hybrid network [24,25,26]. The gelatin–chitosan–5-Fu/ZnO nanoparticle film showed the Zn-O characteristic peak at 654 cm^−1^, and the remaining peaks were identical to those of the gelatin–chitosan film. Additionally, the shift in the amide I band in the hybrid film suggested a significant interaction between the functional groups of chitosan and gelatin.

**Figure 5:**
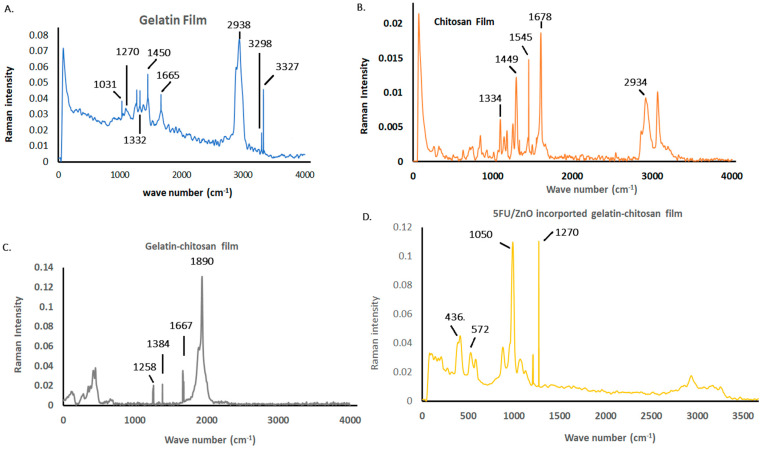
Biofilm characterization is based on the Raman spectra analysis results. (**A**) The results of the gelatin film; (**B**)the chitosan film; (**C**) the chitosan hybrid film; and (**D**) the gelatin–chitosan films containing 5-FU/ZnO.

Figure 5 illustrates the Raman spectra of the prepared film results. The gelatin film exhibits characteristic features, such as the amine III functional group at 1270 cm^−1^, the amide I band at 1665 cm^−1^, and CH_2_ and CH_3_ deformations associated with the 1450 and 2938 cm^−1^ bands, respectively [27]. N-H stretching vibrations are evident at 3327 and 3298 cm^−1^, while CH groups contribute to the absorption band at 1322 cm^−1^. The 1031 cm^−1^ band corresponds to PO_4_^3−^ groups within gelatin molecules. In the chitosan film, the amide I functional group appears at 1678 cm^−1^, with CH deformations observed at 1449 cm^−1^. Additionally, CH_2_ and CH_3_ deformations are linked to the 1545 and 2934 cm^−1^ bands, and the absorption at 1334 cm^−1^ arises from CH_2_ bands of the acetyl group in chitosan [28,29]. Interaction with chitosan molecules shifts the amide III bands of gelatin to 1258 cm^−1^ in the gelatin–chitosan film. The presence of chitosan is confirmed by its characteristic band at 1667 cm^−1^, and the acetyl group of chitosan shifts to 1384 cm^−1^ due to interactions with gelatin molecules [27,28]. N-H stretching vibrations are absent in the hybrid network, indicating their involvement in the hybrid network formation. The amide III bond is observed in the 5-FU/ZnO-integrated gelatin–chitosan film at 1270 cm^−1^. Furthermore, the presence of 5-FU/ZnO introduces peaks at 436 and 572 cm^−1^, while other peaks remain consistent with those of gelatin–chitosan films [28,29]. This suggests strong interactions and the successful integration of 5-FU/ZnO within the film matrix. The shift in the amide I band in the hybrid film further indicates significant interactions between the functional groups of chitosan and gelatin, enhancing the film’s structural integrity and potential functionality.

**Figure 6:**
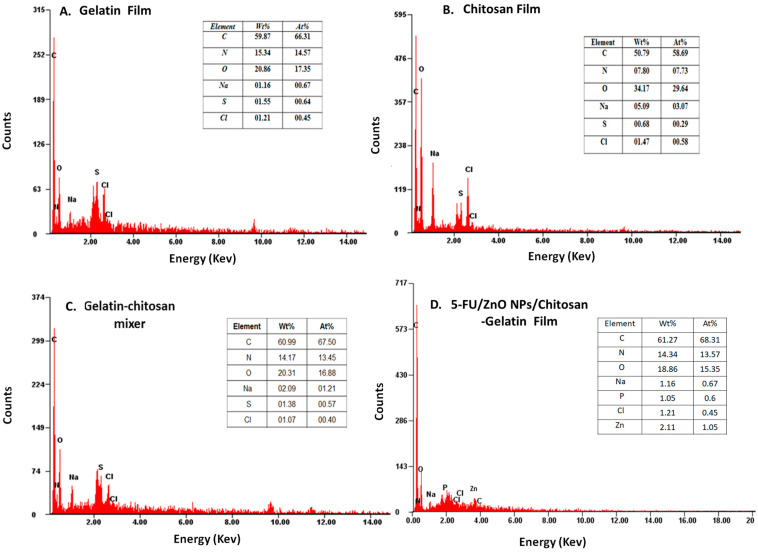
The analysis results for the (**A**) gelatin film, (**B**) chitosan film, (**C**) gelatin–chitosan film, and (**D**) 5-FU/ZnO-integrated gelatin–chitosan films employing EDX spectroscopy.

The elemental composition of the produced film was analyzed using EDX spectra, and the findings are presented in Figure 6, indicating the presence of carbon, oxygen, and nitrogen in different ratios. The gelatin–chitosan film incorporated with 5-FU/ZnO nanoparticles showed distinct Zn ion peaks contributing 1% by weight. This confirms the presence of 5-FU/ZnO nanoparticles within the gelatin–chitosan film.

**Figure 7:**
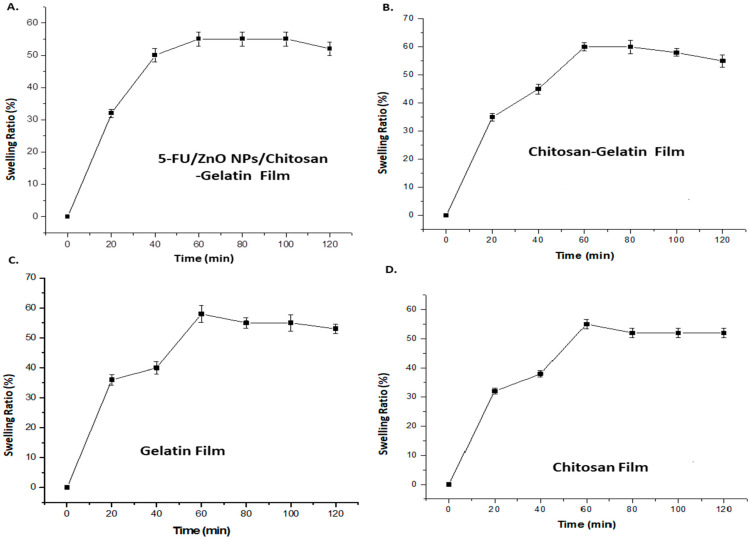
The swelling ratio results for (**A**) 5-FU/ZnO-integrated gelatin–chitosan films; (**B**) gelatin–chitosan films; (**C**) gelatin films; and (**D**) chitosan films.

**Figure 8:**
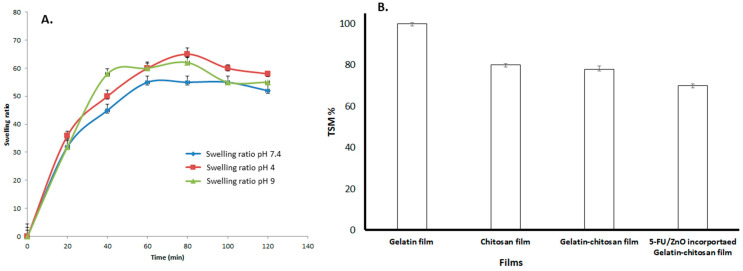
The results of the pH-based swelling ratio and total soluble matrix (%) of films. (**A**) The swelling ratios of 5-FU/ZnO-incorporated gelatin–chitosan was studied at various pH levels (4, 7.4, and 9). (**B**) The total soluble matrices of gelatin, chitosan, gelatin–chitosan, and 5-FU/ZnO-integrated gelatin–chitosan films.

The swelling curve was used to investigate the water-taking capacity of the film after incorporating 5-Fu/ZnO nanoparticles. In this study, PVA was utilized as a stabilizer, which can alter both the physical properties of the polymer and the film qualities. The polymers containing 5-FU/ZnO particles also increase the cross-linking agent. Polyvalent cations, such as 5-FU/ZnO, act as bridges between the polymer chains of chitosan and gelatin, creating a junction zone that forms the hybrid network [29,30]. The degree of cross-linking and synergy between the two biopolymers have a direct impact on the physicochemical properties of the resultant films, particularly their barrier qualities and water behavior. So, we tested the swelling ratio at various time intervals, and the results are presented in Figure 7. According to the findings, the hybrid film has a maximum water absorption capacity of 61%, while the 5-FU/ZnO-incorporated gelatin–chitosan film has a capacity of 56%, indicating that the 5-FU/ZnO nanoparticles also contributed to the construction of the film network and the formation of a more stable polymer network. Less water molecules can permeate the sample, reducing swelling [31]. The 5-FU/ZnO NP-integrated film swelling ratio was tested in three different pH solutions, and the results are shown in Figure 8A; the other parameters were kept constant (5.5 cm diameter). The temperature was 25 °C, and the relative humidity was −75%. Aluminum containers (with dimensions height h = 8cm; outer diameter d = 5.5 cm; and inner diameter d = 5 cm) filled with desiccant (anhydrous CaCl_2_) were coated with samples of the films (diameter 5.5 cm) attached by a thin layer of paraffin wax and topped with a plastic ring [29,30]. The results revealed that the highest swelling rate was 62% at pH 9.0 and around 50% at pH 4, but at pH 7.4, the maximum value was around 58% after 60 min, following which the values started decreasing [30,31]. To estimate dry maters, total soluble mater investigations (TSM) were performed, and the results are shown in Figure 8B. The films (2 × 2 cm, weighing 70 mg) were chocked in milli-Q-water at pH = 7 for 24 h at room temperature (27 °C) and then dried at 105 °C. The undissolved film’s weight was then estimated. The results confirmed that the gelatin film showed 100% total soluble matter; chitosan showed 80% ± 0.5% soluble matter; the gelatin–chitosan film showed 78% ± 1.5% soluble matter; and the 5-Fu/ZnO-integrated film showed 70 ± 0.7% soluble matter.

The in vitro drug release rate was examined at pH 7.4, and the maximum percentage (%) of drug release was estimated over 24 h using the linear equation Y = 0.048x + 0.0098Y = 0.048x + 0.0098Y = 0.048x + 0.0098. A maximum of 72.5% of 5-FU was released from the film. The encapsulating material matrix, release medium, and drug molecule significantly impact drug release. The graph was created using a linear regression fit to analyze the drug release kinetics. The linear curve of cumulative drug release vs. time was plotted for the zero-order kinetic model. The log cumulative drug remaining vs. time plot was used to examine the first-order kinetic model. The Higuchi model was assessed by plotting cumulative drug release vs. the square root of time, while the Korsmeyer–Peppas model was evaluated by plotting log cumulative drug release vs. log time [30,31]. The Hixson–Crowell model was tested by graphing the cube root of the drug remaining vs. time.

**Figure 9:**
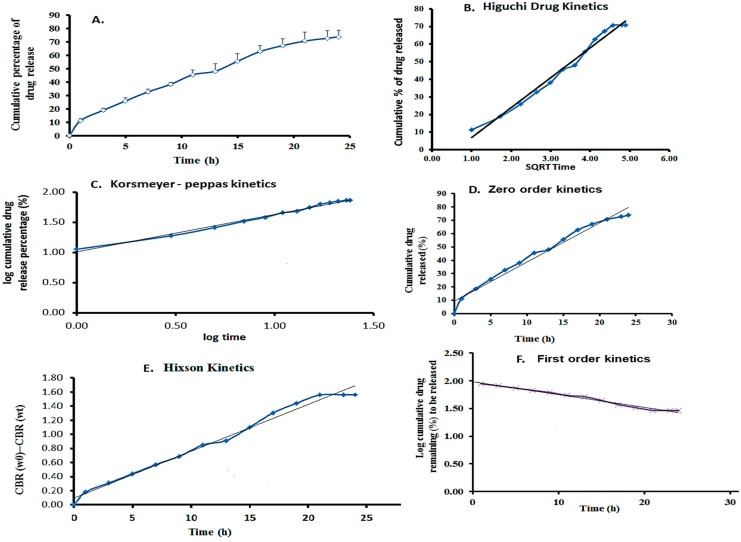
Drug release studies. (**A**) Results for the cumulative percentage of drug release. (**B**) Results of the Higuchi kinetics model. (**C**) Results of the Korsmeyer–Peppas kinetics model. (**D**) Zero-order kinetics model. (**E**). Hixon kinetics model. (**F**) First-order kinetics model.

The results showed that the hydrophilic medication 5-FU release closely followed the Korsmeyer–Peppas model. This model revealed a non-Fickian drug release mechanism from the created nanoparticles’ incorporated film, as well as the percentage of 5-FU released from the films based on the erosion, swelling, and dissolution of the nanoparticle and film layers. The kinetics demonstrated that the cylindrical structure of β-CD, nanoparticles, and film composition had a direct impact, producing delayed drug release profiles. These particles could also be incorporated into a film for easier application, as shown in Figure 9. Table 1 compares the R^2^ values for different drug kinetics models, illustrating the fit of each model to the experimental data. This table highlights which model best describes the drug’s absorption and elimination behavior.

**Figure 10:**
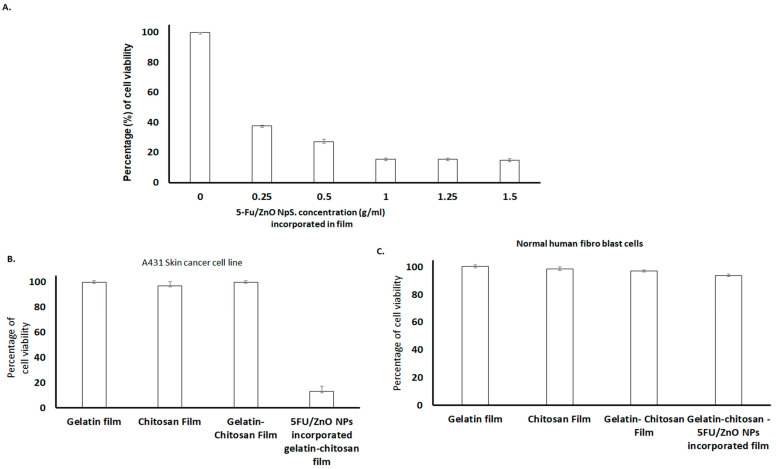
Results of cytotoxic studies. (**A**) Different concentrations of 5-Fu/ZnO-incorporated gelatin–chitosan films (0~1.5 g/mL) on A431 skin cancer cell lines. (**B**) Specific cancer cell-killing efficiency comparison of the films. The results clearly indicated that the 5-FU/ZnO-incorporated film showed a maximum of 85% of cancer cell killing (**C**) and gelatin film, chitosan film, gelatin–chitosan film and 5-Fu/ZnO-incorporated film against normal cells. The relative cell viability read for the control after 72 h of incubation was taken as the reference (100%). Data are expressed as the mean ± SEM of five separate experiments. Significance was calculated by a one-way ANOVA.

According to these findings, the 5-FU–βCD/ZnO nanoparticle-incorporated film exhibited a significant decrease in cell viability percentage compared to the plain film. Initially, different concentrations of 5-FU/ZnO nanoparticles were mixed with the film, and their impact on cell viability was assessed in cancer cells. The results depicted in Figure 10 demonstrate that various films, including gelatin, chitosan, gelatin–chitosan, and 5-FU/ZnO NP-incorporated gelatin–chitosan films, were all fully biocompatible. Normal healthy cells exhibited no change in viability, affirming the safety of the anti-cancer medication-embedded biofilm for normal cells. Specifically, the presence of 5-FU/ZnO nanoparticles led to cancer cell death, facilitated by the biofilm’s ability to protect the nano-drug from leakage, ensure controlled drug release, and enhance entry into target cells. This property is crucial in cancer treatment, enhancing therapeutic efficacy and minimizing side effects, thus highlighting the film’s potential for cancer therapy [32]. Furthermore, the absence of preservatives and solvents in these film formulations mitigates the risk of skin irritation.

This section summarizes the results of cytotoxic experiments conducted on skin cancer cell lines and normal human fibroblast cells, as detailed in Figure 10. Significant changes in cell viability were observed with increasing concentrations of 5-FU/ZnO nanoparticles compared to their respective zero concentrations. According to the dataset in Figure 10A, at a concentration of 1 g/mL, 5-FU/ZnO NPs achieved a maximal killing efficiency of 84.43 ± 1.5%, with similar results observed at 1.5 g/mL and 2 g/mL, indicating that 1 g/mL is sufficient for developing an effective anti-cancer film. The relative histogram dataset illustrates the cell viability response of the A431 skin cancer cell line compared to normal human fibroblasts [32]. In the A431 skin cancer cell line, 5-FU/ZnO NPs incorporated into a gelatin–chitosan film demonstrated a cell viability reduction of 15.66 ± 0.56%, indicating pronounced therapeutic efficacy. These beneficial effects are more pronounced in skin cancer cell lines (Figure 10B) compared to normal human fibroblast cells (Figure 10C). Statistical analysis using IBM SPSS revealed a significant ANOVA result (*p* < 0.05) across the experimental data, highlighting the greater impact on cancer cell viability compared to normal cells, which exhibited less than 5% impact [32]. The integration of 5-FU/ZnO NPs at a concentration of 1 g/mL in the biofilm demonstrates higher killing efficiency against cancer cells.

## 4. Conclusions

In this study, a multifunctional biopolymer film was efficiently developed, incorporating 5-FU-conjugated ZnO nanoparticles into the biofilm matrix. Analysis via SEM, FT-IR, EDX, and Raman spectroscopy confirmed the uniformity of the film’s structure and the successful incorporation of 5-FU/ZnO nanoparticles. Hydration studies demonstrated the film’s ability to adhere effectively to the skin. Furthermore, the anticancer efficacy of the 5-FU/ZnO nanoparticles encapsulated within the biopolymer film was evaluated against skin cancer cells, revealing significant anticancer activity while also maintaining safety for normal cells. This finding underscores the potential of gelatin–chitosan films containing 5-FU/ZnO nanoparticles as promising candidates for future applications as skin patches in the treatment of skin cancer. To ensure their safety and efficacy for clinical use, further investigations should focus on the biocompatibility and long-term stability of these films. Additionally, research could explore the optimization of drug release kinetics and the potential synergistic effects of combining 5-FU/ZnO nanoparticles with other therapeutic agents for enhanced anticancer activity. Overall, this study paves the way for developing innovative and effective approaches for skin cancer treatment using advanced biopolymer-based formulations.

## Figures and Tables

**Table 1:** The drug kinetics table displayed each model’s R^2^ values.

Film	Drug Kinetics Model	R^2^ Values
5-FU/ZnO-incorporated Gelatin–chitosan film	Higuchi (B)	0.9851
	Korsmeyer–Peppas (C)	0.9916
	Hixson (E)	0.9865
	Zero-order (D)	0.9743
	First-order (F)	0.9861
Order of R^2^ values for models	(C) < (E) < (F) < (B) < (D)	

## Data Availability

Dataset available on request from the authors.
